# Association of non-high-density lipoprotein cholesterol to high-density lipoprotein cholesterol ratio (NHHR) with 90-day mortality in acute pancreatitis: A MIMIC-IV database analysis

**DOI:** 10.1371/journal.pone.0343716

**Published:** 2026-02-23

**Authors:** Jiali Xu, Jing Long, Gang Luo, Mingming Deng, Liang Wang

**Affiliations:** 1 Department of Gastroenterology, The Affiliated Hospital of Southwest Medical University, Luzhou, Sichuan, China; 2 Department of Gastroenterology, The People’s Hospital of Leshan, Leshan, Sichuan, China; 3 Department of Gastroenterology, Chongqing University Jiangjin Hospital (Jiangjin Central Hospital), Chongqing, China; 4 Department of Emergency Medicine, The Affiliated Hospital of Southwest Medical University, Luzhou, Sichuan, China; Northwestern University Feinberg School of Medicine, UNITED STATES OF AMERICA

## Abstract

The non-high-density lipoprotein cholesterol to high-density lipoprotein cholesterol ratio (NHHR) is a new composite lipid index. Blood lipid levels are closely associated with the severity of acute pancreatitis (AP), but the relationship between NHHR and mortality in AP patients remains unclear. Therefore, the purpose of this study is to investigate the connection between NHHR and the mortality of patients with AP within 90 days. A total of 454 adult participants with AP from the MIMIC-IV database were categorized into three groups based on their NHHR levels. We employed a multivariate Cox proportional hazards model to assess the relationship between NHHR and 90-day mortality in AP patients. Additionally, we utilized the random forest method to identify the ten most significant risk factors associated with mortality in AP, which was subsequently used to build a prediction model. Out of the patients with AP, 27 died within 90 days. After adjusting for various factors, the hazard ratios for mortality across the tertiles of the NHHR (from the lowest to the highest tertiles: Q1–Q3) were as follows: 1.00 (reference), 1.47 (95% CI: 0.47–4.66), and 4.07 (95% CI: 1.43–11.60). Additionally, the area under the curve (AUC) for our AP mortality prediction model is 0.867 (95% CI: 0.769–0.964). Our findings indicate that a high level of NHHR is closely associated with the mortality of AP, serving as a simple index to predict the prognosis of AP patients.

## Introduction

Acute pancreatitis (AP) is a common condition seen in clinical settings, with an incidence rate of 3,374 cases per 100,000 people [1]. Following an initial episode of AP, approximately 21% of patients experience a recurrence, while 36% may go on to develop chronic pancreatitis [2]. Although most cases are mild, around 20% of patients progress to severe or necrotizing pancreatitis, and the mortality rate associated with AP due to local and systemic complications can reach as high as 20–30% [3,4]. In recent years, there has been an increase in cases of AP linked to hypertriglyceridemia (HTG). This rise may be closely associated with various pathological effects of free fatty acids, mechanisms of inflammatory responses, involvement of microcirculation, serum calcium overload, oxidative stress, endoplasmic reticulum dysfunction, genetic polymorphisms, and changes in intestinal microflora [5].

In the current study, an increase in serum triglyceride levels was an independent predictor of worsening acute pancreatitis severity [6]. The strong correlation between cholesterol levels and mortality is significant for predicting the outcomes of patients with acute pancreatitis [7]. The non-high-density lipoprotein cholesterol to high-density lipoprotein cholesterol ratio (NHHR), a new comprehensive blood lipid index, has emerged as an important factor in the development of various metabolic diseases, including diabetes, cardiovascular diseases, non-alcoholic fatty liver disease, hyperuricemia, and gallstones [8–14].

Research indicates that the concentration of dyslipidemia markers is closely linked to the incidence and mortality rates of AP [7]. However, existing studies have not explored the potential role of the NHHR in AP mortality. The correlation between NHHR and AP is not well-studied, and its clinical significance requires clarification through investigations with larger sample sizes. Therefore, this study aims to explore the relationship between NHHR and the risk of death from AP, ultimately providing more accurate evidence for prognosis assessment and personalized treatment in clinical practice.

## Methods

### Data extraction

**Fig 1:**
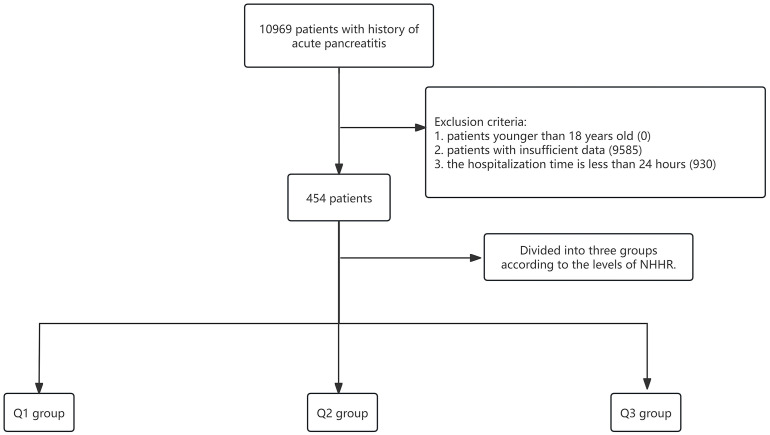
Flow chart.

This retrospective study examined health-related data sourced from the Medical Information Mart for Intensive Care-IV (MIMIC-IV) database [15] (https://doi.org/10.13026/kpb9-mt58; https://physionet.org/content/mimiciv/3.1/). We have completed the necessary MIMIC training (Record ID: 46961999) to gain authorized access to the data. Patients diagnosed with AP were included based on the criteria established in the 9th and 10th editions of the International Classification of Diseases. The exclusion criteria were as follows: (1) Patients younger than 18 years; (2) Patients with insufficient data regarding cholesterol and high-density lipoprotein on the first day of admission; (3) Hospitalization durations of less than 24 hours. In total, 454 patients were included in the study and categorized into three groups based on the type of NHHR (see Fig 1). Data collected from the MIMIC-IV database within the first 24 hours of admission included various patient demographics and clinical information. This data encompassed gender, age, marital status, race, smoking history, and alcohol consumption history, as well as details on medical conditions such as acute kidney injury (AKI), sepsis, myocardial infarction (MI), congestive heart failure (CHF), cerebrovascular diseases (CVD), hypertension, diabetes, and cancer history. Information about liver disease, invasive procedures, cardiopulmonary resuscitation (CPR), and continuous renal replacement therapy (CRRT) was also collected. In addition, laboratory values were recorded, including heart rate (HR), white blood cell count (WBC), neutrophils, hematocrit, platelet count, albumin level, creatinine, calcium, prothrombin time (PT), international normalized ratio (INR), alanine aminotransferase (ALT), aspartate aminotransferase (AST), alkaline phosphatase (ALP), total bilirubin (TBIL), blood amylase (AMY), blood glucose levels, potassium, and sodium. The NHHR was then calculated. The MIMIC database anonymizes information by substituting patient identifiers with randomized codes, which exempts it from requiring individual consent or ethical approval.

### Clinical outcome

The main objective of this study was to assess all-cause mortality within 90 days.

### Statistical analysis

Continuous variables are presented as means with standard deviations, and categorical variables are expressed as frequencies and percentages. Associations between the NHHR index and the endpoint were evaluated using Cox proportional hazards regression (survival package, version 3.8–3) with three models: Model 1, unadjusted; Model 2, adjusted for age and gender; and Model 3, adjusted for age, gender, and congestive heart failure. To assess the robustness of the final Cox model, we conducted stratified bootstrap (boot package, version 1.3–31) resampling by event status. From the original dataset, 1,000 bootstrap samples were drawn with replacement; Model 3 was refit in each sample, and the hazard ratio (HR) for the NHHR index and its 95% confidence interval (CI) were recorded.

In this study, we developed a random forest (RF) classifier to predict 90-day mortality in patients with AP using the randomForest package (version 4.7–1.2). The dataset was randomly partitioned into training (70%) and test (30%) subsets. The model was fitted on the training set and evaluated on the held-out test set using receiver operating characteristic (ROC) analysis and the area under the curve (AUC). To address class imbalance, we applied resampling with the caret package (version 7.0−1), retrained the RF on the resampled training data, and generated a new ROC curve to further assess performance robustness.

Shapley’s Additive Explanations (SHAP) analysis enhances the scalability of the proposed model by utilizing the shapviz package (version 0.9.7). To evaluate the global impact of each feature, we calculate the average SHAP value for each input. These values are then organized in descending order of importance, accompanied by a chart that displays the SHAP values. In the SHAP diagram, each raw material is represented by a point. The x-axis shows the SHAP value, while the y-axis indicates the importance of the features. A higher position on the y-axis represents a greater influence of that characteristic on the output. The importance is illustrated using a color gradient that ranges from light to dark. Furthermore, the SHAP diagram highlights the interactions between features and their effects on the results, employing a color scheme to emphasize these interactions.

We conducted our statistical analysis using RStudio with R version 4.4.2. To complete the demographic characteristic data, we applied the multiple imputation method using the mice package (version 3.18.0). We developed random forest models using the randomForest package (version 4.7–1.2), while the pROC package (version 1.18.5) was utilized to perform ROC analysis and evaluate the area under the curve (AUC). A p-value of less than 0.05 was deemed statistically significant.

## Results

### Characteristics of study participants

A total of 454 participants were involved in this study, with an average age of 55 years and a male representation of 57%. Table 1 outlines the baseline characteristics of the participants based on their NHHR tertiles. Those with higher NHHR scores tended to be younger and predominantly male compared to participants in the lowest tertiles. Additionally, in the higher NHHR group, there was a marked increase in the number of individuals with AKI, sepsis, alcohol-related issues, invasive procedures, and those requiring CRRT therapy. This group also exhibited higher levels of platelets, ALP, TBIL, and glucose. Conversely, the levels of albumin, calcium, PT, and sodium were found to be lower (see Table 1).

**Table 1:** Characteristics of study participants.

	Overall	NHHR (Q1)	NHHR (Q2)	NHHR (Q3)	*P*
N	454	151	148	155	
Female,n(%)	195 (43.0)	74 (49.0)	64 (43.2)	57 (36.8)	0.096
Age,years(mean (SD))	55.34 (17.37)	58.95 (17.75)	56.39 (18.23)	50.84 (15.15)	<0.001
Marital,n (%)					0.235
Single	180 (39.6)	55 (36.4)	53 (35.8)	72 (46.5)	
Married	191 (42.1)	67 (44.4)	67 (45.3)	57 (36.8)	
Divorced	40 (8.8)	14 (9.3)	10 (6.8)	16 (10.3)	
Widowed	34 (7.5)	13 (8.6)	15 (10.1)	6 (3.9)	
Unknown	9 (2.0)	2 (1.3)	3 (2.0)	4 (2.6)	
Race,n (%)					0.007
White	310 (68.3)	99 (65.6)	98 (66.2)	113 (72.9)	
Black	74 (16.3)	35 (23.2)	22 (14.9)	17 (11.0)	
Others	64 (14.1)	17 (11.3)	27 (18.2)	20 (12.9)	
Unknown	6 (1.3)	0 (0.0)	1 (0.7)	5 (3.2)	
Mortality,n(%)	27 (5.9)	5 (3.3)	7 (4.7)	15 (9.7)	0.047
Smoking,n (%)	10 (2.2)	6 (4.0)	1 (0.7)	3 (1.9)	0.146
Alcohol,n (%)	6 (1.3)	1 (0.7)	0 (0.0)	5 (3.2)	0.033
AKI,n(%)	77 (17.0)	19 (12.6)	20 (13.5)	38 (24.5)	0.008
Sepsis,n(%)	61 (13.4)	13 (8.6)	15 (10.1)	33 (21.3)	0.002
MI,n(%)	36 (7.9)	12 (7.9)	13 (8.8)	11 (7.1)	0.863
CHF,n(%)	51 (11.2)	18 (11.9)	22 (14.9)	11 (7.1)	0.096
CVD,n(%)	33 (7.3)	13 (8.6)	13 (8.8)	7 (4.5)	0.266
Hypertension,n(%)	200 (44.1)	68 (45.0)	67 (45.3)	65 (41.9)	0.807
Diabetes,n(%)	132 (29.1)	37 (24.5)	39 (26.4)	56 (36.1)	0.055
Cancer,n(%)	21 (4.6)	5 (3.3)	9 (6.1)	7 (4.5)	0.520
Liver disease,n(%)	22 (4.8)	4 (2.6)	9 (6.1)	9 (5.8)	0.304
Invasive puncture,n(%)	45 (9.9)	10 (6.6)	11 (7.4)	24 (15.5)	0.016
CPR,n(%)	1 (0.2)	0 (0.0)	1 (0.7)	0 (0.0)	0.355
CRRT,n(%)	15 (3.3)	2 (1.3)	3 (2.0)	10 (6.5)	0.025
HR,bpm(mean (SD))	96.10 (11.53)	96.16 (8.42)	95.30 (11.49)	96.80 (13.97)	0.525
WBC, K/UL(mean (SD))	10.28 (5.93)	10.42 (7.12)	9.44 (4.41)	10.94 (5.86)	0.083
Neutrophils,K/uL(mean (SD))	8.04 (4.97)	7.74 (4.82)	7.64 (4.65)	8.71 (5.35)	0.116
Hematocrit,%(mean (SD))	35.82 (6.02)	36.56 (5.64)	35.67 (5.97)	35.23 (6.39)	0.145
Platelet,K/uL(mean (SD))	239.58 (115.29)	224.46 (90.48)	234.94 (107.01)	258.75 (139.92)	0.028
Albumin,g/dLL(mean (SD))	3.59 (0.65)	3.78 (0.62)	3.63 (0.63)	3.36 (0.64)	<0.001
Creatinine,mg/dL(mean (SD))	1.26 (1.50)	1.17 (1.36)	1.30 (1.55)	1.31 (1.58)	0.665
Calcium,mmol/L(mean (SD))	8.52 (0.87)	8.60 (0.71)	8.72 (0.71)	8.25 (1.07)	<0.001
PT(mean (SD))	15.74 (8.77)	15.01 (6.69)	17.41 (12.92)	14.86 (4.42)	0.018
INR(mean (SD))	1.43 (0.87)	1.31 (0.61)	1.63 (1.31)	1.34 (0.40)	0.003
ALT,IU/L(mean (SD))	129.20 (554.32)	95.15 (166.10)	201.94 (942.18)	92.91 (148.55)	0.151
AST,IU/L(mean (SD))	206.60 (1285.42)	80.21 (114.38)	460.11 (2226.14)	87.66 (159.68)	0.014
ALP,IU/L(mean (SD))	136.94 (165.76)	128.78 (139.52)	111.28 (108.51)	169.40 (221.06)	0.007
TBIL,mg/dL(mean (SD))	1.59 (3.31)	1.13 (2.09)	1.51 (3.25)	2.11 (4.17)	0.032
Amylase,IU/L(mean (SD))	436.81 (309.18)	443.89 (283.44)	448.95 (269.30)	418.34 (364.95)	0.651
Glucose,mg/dL(mean (SD))	133.58 (88.09)	137.03 (116.78)	117.68 (53.07)	145.39 (80.38)	0.019
Potassium,mEq/L(mean (SD))	4.06 (0.71)	4.06 (0.73)	4.09 (0.78)	4.02 (0.63)	0.712
Sodium,mEq/L(mean (SD))	137.90 (4.17)	138.52 (3.70)	138.30 (4.02)	136.92 (4.56)	0.001

NOTE: NHHR -the non-high-density lipoprotein cholesterol to high-density lipoprotein cholesterol ratio, AKI -acute kidney injury, MI -myocardial infarction, CHF -congestive heart failure, CVD -cerebrovascular diseases, CPR -cardiopulmonary resuscitation, CRRT -continuous renal replacement therapy, HR -heart rate, WBC -white blood cells, PT -prothrombin time, INR -international normalized ratio, ALT -alanine aminotransferase, AST -aspartate aminotransferase, ALP -alkaline phosphatase, TBIL -total bilirubin

### Relationship between NHHR and mortality

A total of 27 patients with AP died. The relationship between the NHHR and mortality due to AP is presented in Table 2. We utilized the Cox proportional hazards regression model to estimate the independent association between NHHR and the risk of death. After adjusting for multiple variables (Model 3), the hazard ratios (HR) and 95% confidence intervals (CI) for mortality across the lowest to highest tertiles of NHHR (Q1–Q3) were as follows: 1.00 (reference), 1.47 (95% CI: 0.47–4.66), and 4.07 (95% CI: 1.43–11.60), respectively. Trend analysis indicated a linear relationship between NHHR and mortality from AP (trend = 0.009), as shown in Table 2. Due to the limited number of events, we conducted a Bootstrap verification, which yielded an HR of 4.27 (95% CI: 1.34–14.91). The bootstrap validation yielded an HR of 4.27 (95% CI: 1.34–14.91), closely aligning with the original Model 3 estimate (HR = 4.07).

**Table 2:** Cox proportional hazards regression analysis of the relationship between NHHR and the risk of AP-related death.

	Model 1		Model 2		Model 3	
	HR (95% CI)	p	HR (95% CI)	p	HR (95% CI)	p
NHHR						
Q1	1		1		1	
Q2	1.44(0.46, 4.54)	0.533	1.54(0.49, 4.86)	0.462	1.47 (0.47,4.66)	0.509
Q3	3.06(1.11, 8.42)	0.030	3.98(1.40, 11.30)	0.010	4.07(1.43, 11.60)	0.009
P for trend	0.030		0.011		0.009	

Model 1 was unadjusted.

Model 2 was adjusted for age and gender.

Model 3 was adjusted for age, gender, and CHF.

CHF – congestive heart failure.

### Random forest model

**Fig 2:**
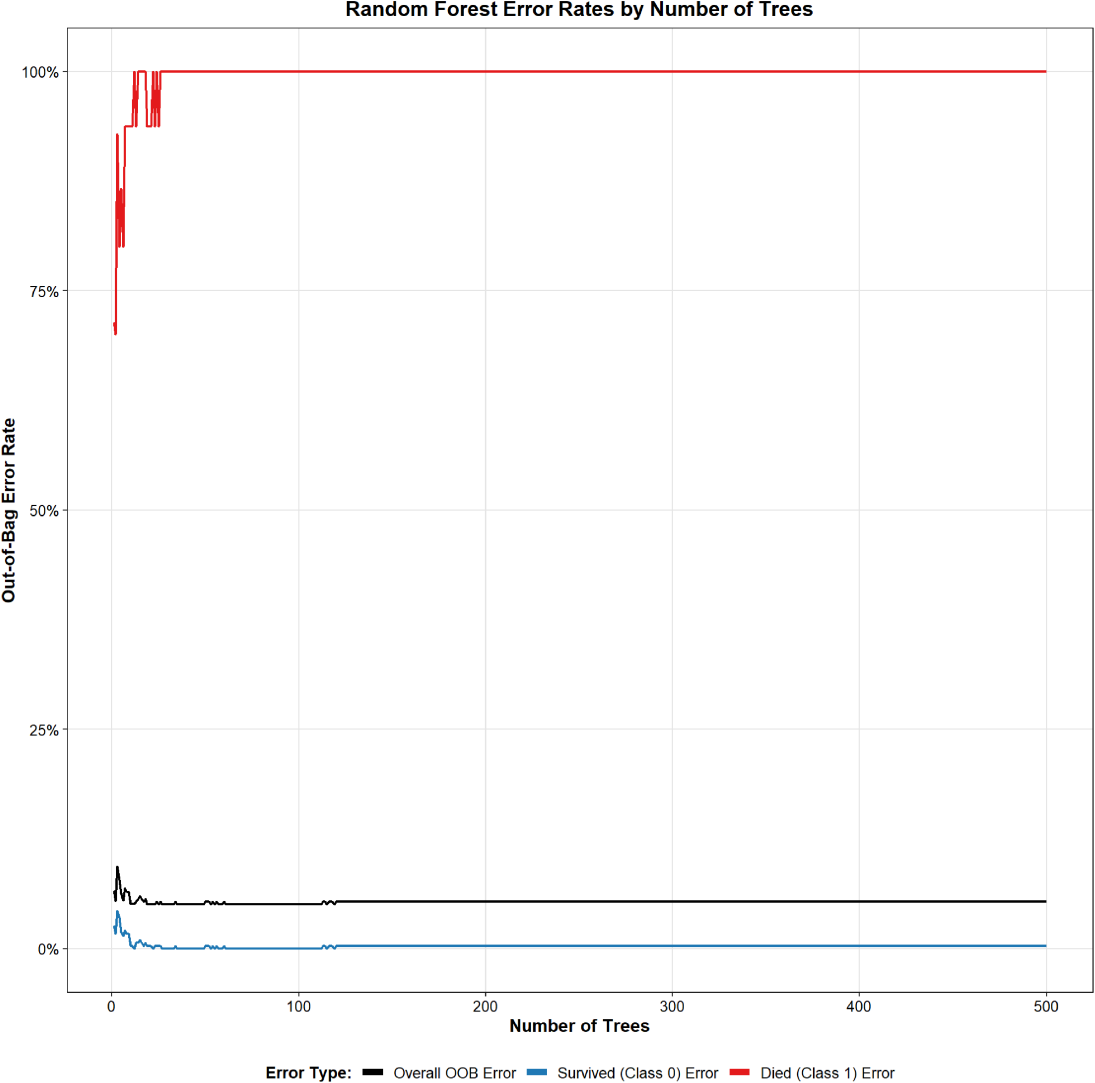
Error Map for the random forest model.

**Fig 3:**
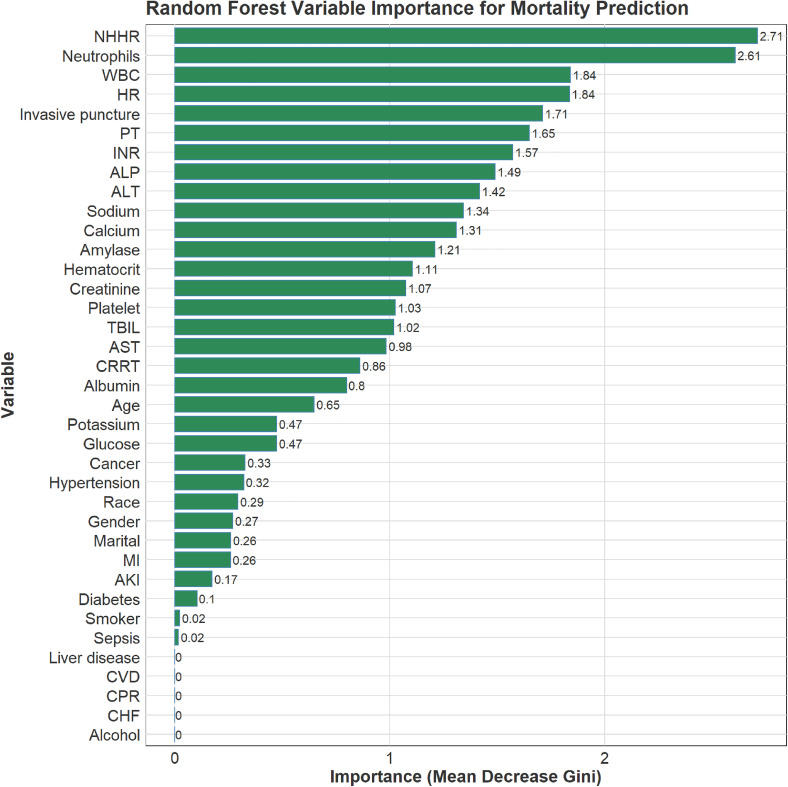
Importance of features in a random forest classifier. NOTE: NHHR -the non-high-density lipoprotein cholesterol to high-density lipoprotein cholesterol ratio, WBC -white blood cells, HR -heart rate, PT -prothrombin time, INR -international normalized ratio, ALP -alkaline phosphatase, ALT -alanine aminotransferase, TBIL -total bilirubin, AST -aspartate aminotransferase, CRRT -continuous renal replacement therapy, MI -myocardial infarction, AKI -acute kidney injury, CVD -cerebrovascular diseases, CPR -cardiopulmonary resuscitation, CHF -congestive heart failure.

**Fig 4:**
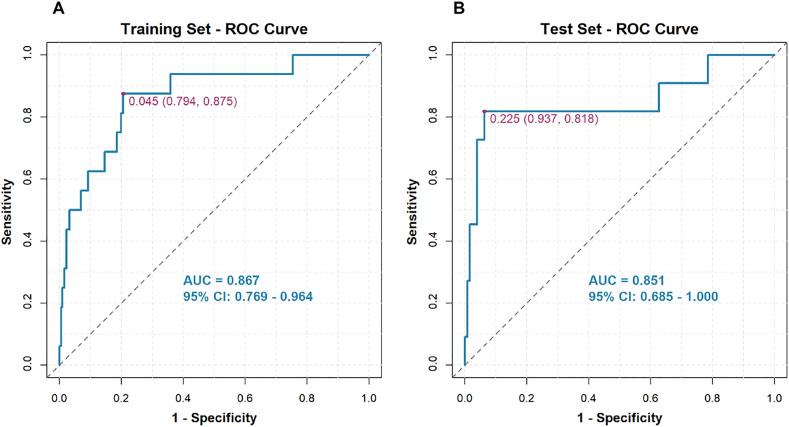
ROC curve for the AP mortality prediction model, showing results for both the training cohort (A) and the validation cohort (B).

**Fig 5:**
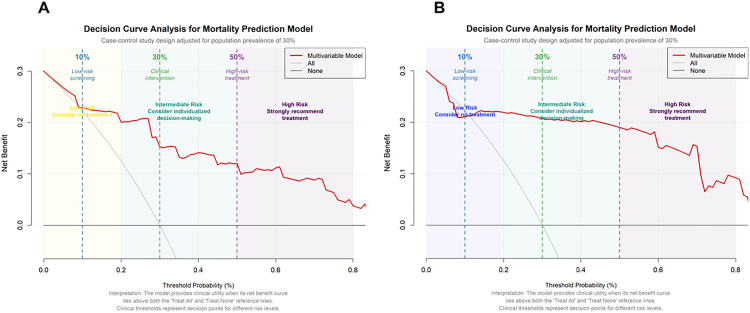
DCA Curve for the AP mortality prediction model (A: Training Cohort, B: Validation Cohort).

**Fig 6:**
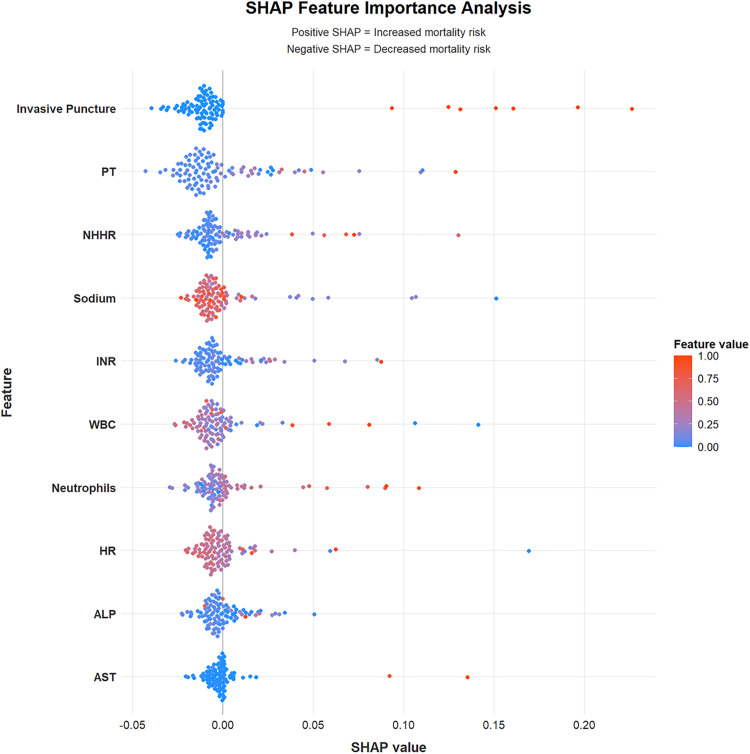
SHAP-based feature analysis demonstrating. NOTE: The plot displays the relative importance and contribution of each variable in the predictive model. Features are ranked vertically based on their mean absolute SHAP value, which indicates their overall significance. Each point on the plot represents an individual observation; its horizontal position reflects the SHAP value (positive values increase predicted risk, while negative values decrease predicted risk). The color of each point corresponds to the original feature value, with red indicating high values and blue indicating low values, as illustrated in the color bar. PT -prothrombin time, NHHR -the non-high-density lipoprotein cholesterol to high-density lipoprotein cholesterol ratio, INR -international normalized ratio, WBC -white blood cells, HR -heart rate, ALP -alkaline phosphatase, AST -aspartate aminotransferase.

An RF model was developed to predict mortality in patients with acute pancreatitis (AP). The cohort of 454 patients was randomly split into a training set (n = 317) and a validation set (n = 137). Hyperparameter tuning identified mtry = 6 and ntree = 310 as optimal, yielding the lowest error rate. Variable-importance rankings are shown in Figs 2 and 3. The prediction model was built using the 10 most important predictors and evaluated with ROC analysis. In the training set, the AUC was 0.867 (95% CI, 0.769–0.964; Fig 4A), and the corresponding DCA is presented in Fig 5A. Validation on the held-out set yielded an AUC of 0.851 (95% CI, 0.685–1.000; Fig 4B), with the DCA shown in Fig 5B. Given the limited number of outcome events (27 deaths), we applied resampling to mitigate class imbalance and reassess performance; as shown in Fig 6, the AUC decreased from 0.867 to 0.765 after resampling (Supplementary figure 1).

### SHAP machine learning

SHAP analysis is utilized to explain the predictions of the best-performing random forest (RF) model. This analysis highlights the influence of ten key characteristics on the death prediction model for patients with acute pancreatitis, ranking them according to their SHAP values. Fig 6 provides a comprehensive visualization of the multidimensional impact of each feature, and the SHAP values demonstrate how both high and low levels of these variables affect the overall prediction model. Among these features, Invasive puncture exhibits the strongest predictive ability; an increase in this score significantly elevates the risk of death. PT level closely follows, contributing substantially to the model, indicating that PT levels correlate with increased risk. Furthermore, the NHHR plays an important role in predictions, with higher NHHR levels associated with a greater risk of death. Other significant factors include sodium, INR, WBC, neutrophils, HR, ALP, and AST.

## Discussion

Our research aims to uncover the relationship between NHHR and the risk of death from AP. After adjusting for the influence of covariates, we found that a high level of NHHR is significantly associated with a 90-day mortality risk in AP patients, with an HR of 4.07 when compared to low NHHR levels. We identified ten risk factors for death from AP, including NHHR, and developed a prediction model that demonstrates strong predictive value for mortality in AP cases.

In a study of 238 patients with AP, only 2.22% of those with mild cases died, while the mortality rate for severe cases was significantly higher at 45.63%. Factors such as blood glucose levels, urea, oxygen partial pressure, white blood cell count, hemoglobin, total bilirubin, and cholesterol were closely linked to mortality [7]. A retrospective analysis examined risk factors for death among 74 severe acute pancreatitis (SAP) patients within 24 hours of admission. Univariate logistic regression analysis identified early shock, pleural effusion, pH level, complications, and the acute physiology and chronic health evaluation (APACHE) score as related to mortality in SAP patients [16]. Research has identified multiple organ dysfunction syndrome (MODS) and pancreatic necrosis affecting over 50% of the pancreas as independent predictors of survival in patients with severe pancreatitis [17]. In critically ill patients, a continuous increase in heart rate (HR) is considered a risk factor for poor prognosis. Studies have evaluated the relationship between continuous HR increase and the 90-day mortality rate of acute pancreatitis patients in the intensive care unit (ICU), revealing that this increase is significantly linked to a lower survival rate [18]. Among 380 patients with acute pancreatitis in the ICU, the overall mortality rate was 23.2%. A prediction model incorporating international normalized ratio (INR), albumin, lactic acid, blood urea nitrogen (BUN), and bilirubin demonstrated an area under the curve (AUC) of 0.93 for predicting 7-day mortality and 0.84 for 30-day mortality [19].

Currently, there is increasing research on the factors related to acute pancreatitis death, but a standardized predictive index for AP mortality has not yet been established. Studies have shown that patients with HTG often experience persistent organ failure more frequently than those with normal triglyceride levels. The incidence of persistent organ failure rises with the severity of HTG, with rates of 17% in normal triglycerides, 30% in mild HTG, 39% in moderate HTG, and 48% in severe to very severe HTG [20]. Additionally, numerous studies indicate that severe HTG-AP is positively correlated with the severity, complications, and overall mortality of pancreatitis [21–23]. One study involving 1,127 patients with acute pancreatitis found that serum levels of apolipoprotein A-I (APOA-I) and high-density lipoprotein cholesterol (HDL-C) were negatively correlated with SAP, with odds ratios of 0.24 and 0.16, respectively [24]. Another investigation focused on the relationship between the lowest HDL-C levels and the worsening of acute pancreatitis in the ICU, revealing a negative correlation between the lowest HDL-C levels and the occurrence of SAP, with a relative risk of 0.897 [25]. The area under the ROC curve for predicting the aggravation of acute pancreatitis based on the lowest HDL-C level was found to be 0.707, with an optimal cutoff value of 0.545 mmol/L. Furthermore, the ratio of HDL-C to low-density lipoprotein cholesterol (LDL-C) in deceased patients with AP was significantly higher than that in survivors. Additionally, the area under the ROC curve for this HDL-C/LDL-C ratio was found to be 0.658 [26]. The ratio of triglycerides to HDL-C is also positively correlated with the severity of HTG-AP, and it can serve as a simple index for assessing the severity of HTG-AP [27].

A new composite lipid index, known as the ratio of non-HDL cholesterol to HDL-C, referred to as NHHR, has demonstrated strong predictive value in assessing the risks of various diseases, including diabetes, cardiovascular diseases, sleep disturbances, and depression [28–33]. However, the prognostic significance of NHHR for patients with AP remains unclear. To address this issue, we conducted a study using the MIMIC database to investigate the relationship between NHHR and mortality among AP patients, aiming to provide valuable insights for disease management. Our findings indicate that a high NHHR is associated with increased mortality in AP patients. While the exact biological mechanism linking NHHR to mortality in AP is still not fully understood, some studies suggest that higher NHHR levels are correlated with more severe sepsis. Additionally, sepsis, characterized by elevated NHHR levels, is associated with higher mortality rates [12]. Moreover, as the number of low-density lipoprotein particles increases, glycosylation and oxidation processes, influenced by free radicals, enhance the permeability of the vascular endothelium and promote macrophage aggregation. This contributes to the gradual formation of atherosclerotic plaques. When these plaques become eroded or ruptured, thrombosis can occur, potentially leading to acute cardiovascular events and resulting in patient mortality [8]. High levels of triglycerides (TG) in the body can be hydrolyzed into free fatty acids, which directly increase the acidity of bodily fluids, leading to acidosis. This process also activates trypsinogen, resulting in damage to pancreatic acinar cells and capillary endothelial cells. Additionally, elevated triglycerides contribute to significant stress on the body. The higher the incidence of systemic inflammatory response syndrome (SIRS), the worse the prognosis for patients [34]. HDL serves multiple protective functions, such as promoting cholesterol reverse transport, providing anti-inflammatory effects, acting as an antioxidant, and protecting the endothelium [35]. Non-HDL-C is closely associated with persistent low-grade inflammation, while low levels of HDL-C indicate a lack of anti-inflammatory and endothelial protective mechanisms. In the case of acute pancreatitis, the presence of a “fragile endothelium” and a “proinflammatory internal environment,” characterized by a high NHHR, can significantly intensify the inflammatory response. This leads to more severe systemic endothelial damage, capillary leakage, and microcirculation failure, ultimately accelerating the progression of multi-organ failure. Therefore, NHHR serves as an effective measure to reveal the underlying pathophysiological factors in patients who are at risk of worsening pancreatitis by quantifying the balance between atherosclerosis and anti-atherosclerotic lipoproteins in the body.

This study has several limitations. First, the population is mainly U.S.-based, which may restrict generalizability to other racial and ethnic groups. Second, the MIMIC-IV database does not include certain potential confounders, particularly lipid-lowering therapy and a comprehensive history of dyslipidemia. Moreover, lipid parameters in this study were measured within 24 hours of disease onset and may have been influenced by the acute-phase response, which can lower HDL-C levels and thus lead to an underestimation of NHHR. This may attenuate the observed associations. Third, as MIMIC-IV centers on intensive care settings, our conclusions primarily apply to patients with severe acute pancreatitis and may not extend to mild cases. Lastly, the limited number of outcome events may have led to an overestimated model performance. Future studies should corroborate NHHR’ s predictive utility in large, multicenter cohorts and incorporate serial lipid assessments to separate acute-phase effects from baseline levels, facilitating a more robust appraisal of its independent prognostic value.

## Conclusion

Our findings indicate that a high level of NHHR is significantly associated with the mortality of acute pancreatitis when compared to a low level of NHHR. This suggests that NHHR could serve as a straightforward index for predicting the prognosis of AP patients in the future. Additionally, our prediction model effectively assesses the risk of death for AP patients in the early stages of the condition, thereby aiding clinical decision-making.

## Supporting information

S1 Fig Model evaluation using resampling. (A) Feature importance (top 15 variables) in the random forest classifier; (B) ROC curve. NOTE: NHHR -the non-high-density lipoprotein cholesterol to high-density lipoprotein cholesterol ratio, ALP -alkaline phosphatase, HR -heart rate, PT -prothrombin time, INR -international normalized ratio, AKI -acute kidney injury, AST -aspartate aminotransferase, WBC -white blood cells.
(TIF)
