# Supplementary figures and images for: Association of non-high-density lipoprotein cholesterol to high-density lipoprotein cholesterol ratio (NHHR) with 90-day mortality in acute pancreatitis: A MIMIC-IV database analysis
Source: PLoS One. 2026 Feb 23;21(2):e0343716. doi: 10.1371/journal.pone.0343716 (PMC12928564; doi:10.1371/journal.pone.0343716)

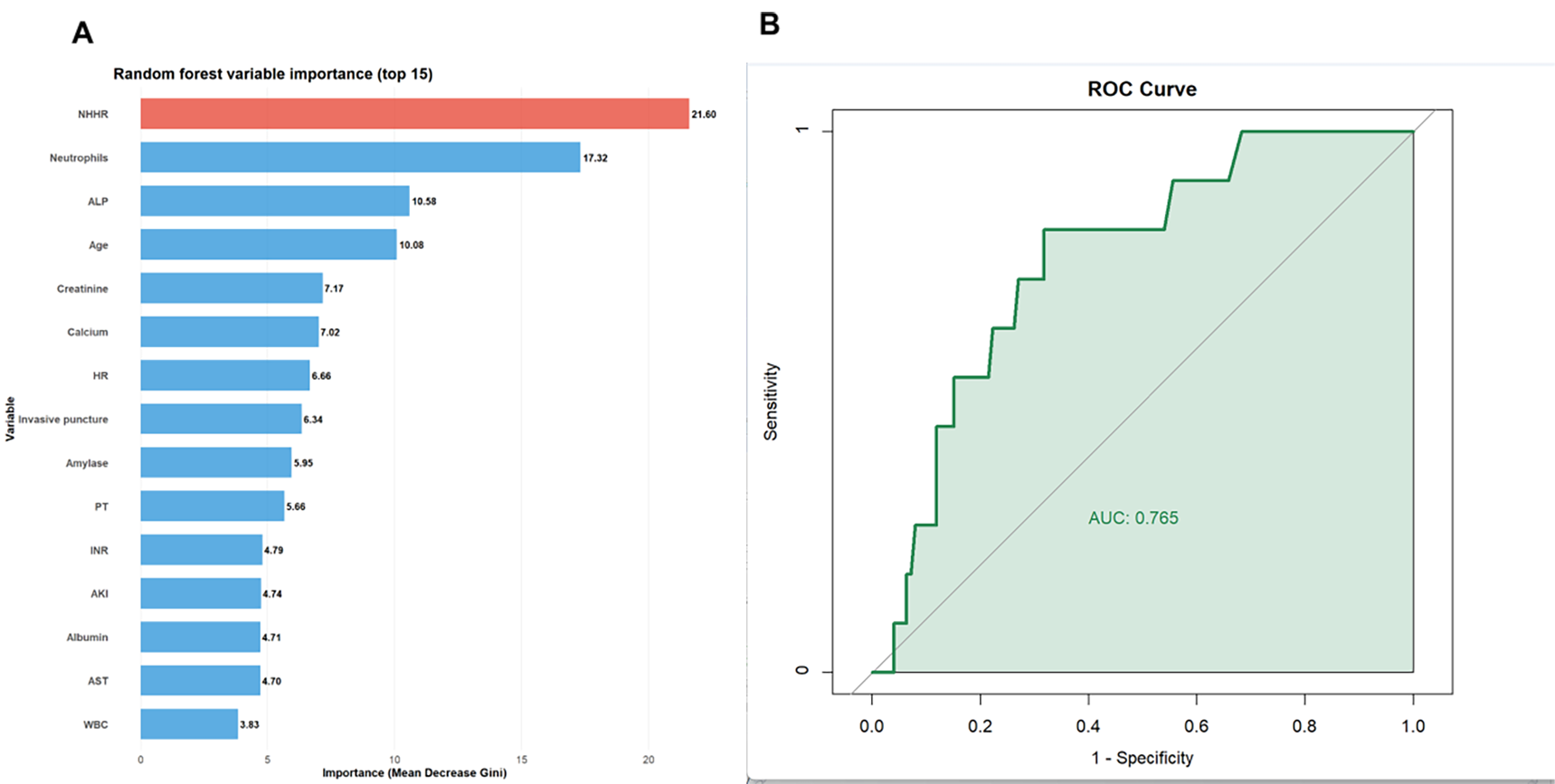

Supplement: S1 Fig — (A) Feature importance (top 15 variables) in the random forest classifier; (B) ROC curve. NOTE: NHHR -the non-high-density lipoprotein cholesterol to high-density lipoprotein cholesterol ratio, ALP -alkaline phosphatase, HR -heart rate, PT -prothrombin time, INR -international normalized ratio, AKI -acute kidney injury, AST -aspartate aminotransferase, WBC -white blood cells. (TIF) [file pone.0343716.s001.tif]
